# Stereotactic radiation therapy for post-transplant systemic recurrence of hepatocellular carcinoma: safety, efficacy and a nomogram predicting overall survival

**DOI:** 10.3389/fonc.2025.1592060

**Published:** 2026-01-21

**Authors:** Jingru Chen, Gengmin Niu, Xin Guo, Yuxuan Tao, Xiaobin Liu, Yan Xie, Li Zhang, Wentao Jiang, Peiguo Wang, Zhongqiu Wang, Zhiyong Yuan, Qingxin Wang

**Affiliations:** 1 Department of Radiation Oncology, Tianjin’s Clinical Research Center for Cancer, Key Laboratory of Cancer Prevention and Therapy, Tianjin Medical University Cancer Institute & Hospital, National Clinical Research Center for Cancer, Tianjin, China; 2 Department of Radiation Oncology, The Affiliated Cancer Hospital of Zhengzhou University & Henan Cancer Hospital, Zhengzhou, China; 3 Department of Radiation Oncology, National Cancer Center/National Clinical Research Center for Cancer/Cancer Hospital of Chinese Academy of Medical Sciences, Langfang, Hebei, China; 4 Department of Radiology, Tianjin Medical Imaging Institute, Tianjin First Central Hospital, School of Medicine, Nankai University, Tianjin, China; 5 Department of Liver Transplantation, Tianjin First Center Hospital, NHC Key Laboratory for Critical Care Medicine, Key Laboratory of Transplantation, Chinese Academy of Medical Sciences, Tianjin, China

**Keywords:** stereotactic radiotherapy, liver transplantation, hepatocellular carcinoma, recurrence, nomogram

## Abstract

**Background:**

Stereotactic Radiation Therapy (SRT) has proven effective for various stages of Hepatocellular Carcinoma (HCC), however, its role in managing intra- or extrahepatic recurrence after liver transplantation remains underexplored.

**Objectives:**

This study evaluates the safety and efficacy of SRT delivered using the CyberKnife^®^ system for recurrent HCC after liver transplantation and introduces a novel nomogram for predicting survival to guide individualized management.

**Methods:**

In a single-center retrospective study conducted between 2007 and 2020, 79 patients with recurrent HCC after transplantation presenting 133 intra- or extrahepatic lesions were treated with SRT. Treatment response, survival outcomes, and local control rates were evaluated. Multivariate analysis identified significant prognostic factors, which were incorporated into a predictive nomogram for survival.

**Results:**

With a median follow-up of 11.4 months, median overall survival (OS) was 15.1 months, and the local control rate was 90.5%. The OS rates at 6 months, 1 year, and 2 years were 78.9%, 57.1%, and 38.9%, respectively. Key factors associated with improved survival included fewer than three lesions, AFP <500 ng/ml, KPS ≥70, and total gross tumor volume (GTV) <40 mL. The nomogram demonstrated good predictive accuracy with a validated C-index of 0.760. Treatment was well tolerated, with no severe treatment-related toxicities observed.

**Conclusion:**

SRT provides effective local control for both intra- and extrahepatic recurrences of HCC after liver transplantation. The proposed nomogram offers a valuable tool for personalized surveillance and treatment planning.

## Introduction

Liver transplantation (LT) offers a potentially curative treatment for selected patients with unresectable hepatocellular carcinoma (HCC) and limited tumor burden. However, post-transplant HCC recurrence occurs in approximately 15-20% of patients, predominantly within the first 2–3 years (1, 2). Immunosuppression post-LT often contributes to aggressive recurrence, characterized by rapid progression, metastasis, and poor median survival (7–16 months) (3).

Effective surveillance serves as the essential entry point to salvage therapies, with multidisciplinary management potentially including resection, ablation, transarterial chemoembolization (TACE), radiation, or systemic agents. Local modalities like stereotactic radiotherapy (SRT) are particularly valuable for controlling oligorecurrence and prolonging survival (4–6).

SRT delivers precise, high-dose radiation (≥5 Gray per fraction [Gy/F]) with sub-centimeter accuracy and motion management. Systems like CyberKnife use real-time tumor tracking and robotic arm–based beam positioning to ensure precise targeting with minimal exposure to surrounding organs (7–10). While SRT demonstrates efficacy for challenging HCC cases (e.g., central location, vascular proximity, inoperability) (11, 12), its application within the post-transplant surveillance pathway - where early detection enables potentially curative salvage - remains poorly characterized.

This study represents the first comprehensive analysis of outcomes using CyberKnife-based SRT for post-transplant HCC recurrence. We also developed a practical prognostic nomogram based on pre-treatment variables available during surveillance to guide personalized salvage therapy after recurrence.

## Methods

### Patient eligibility

From January 2007 to October 2020, SRT was used to treat HCC recurrence in 79 patients with 133 lesions at our institution. Eligibility required confirmed HCC progression, either through biopsy or radiological evidence of tumor growth (≥20% increase in diameter or ≥5 mm growth) per Response Evaluation Criteria in Solid Tumors (RECIST) v1.1 criteria. The decision to administer SRT was reached by multidisciplinary consensus among hepatobiliary transplant surgeons, interventional radiologists, radiation oncologists, and medical oncologists following integrated assessment of lesion anatomy, patient fitness and preferences. This retrospective study was approved by Tianjin Medical University Cancer Institute and Hospital’s Ethics Committee (IRB#: bc2022104).

### Treatment schedule

Patients were positioned supine and immobilized using vacuum bags or thermoplastic masks (brain or cervical spine lesions) for imaging procedures. They underwent Computed Tomography (CT) and, where applicable, Magnetic Resonance Imaging (MRI) for lesions located in the adrenal glands, liver, bone and brain. The imaging was conducted with 1.5mm slice thickness, encompassing a 15cm margin around the tumor. The gross tumor volume (GTV) was outlined and expanded to form the planning target volume (PTV), with margins adjusted based on tumor location, nearby organ risks, and institutional protocols. Thirty-eight lower lung, liver, and adrenal gland targets were treated using the Synchrony Respiratory Tracking System with gold fiducials that were implanted near or inside the treatment target one week prior. Four bone targets near the surface used fiducial tracking, while 91 targets were treated with the skull and X-sight spine-tracking system.

### Follow-up and evaluation

Follow-ups were conducted every 3 months post-SRT, utilizing contrast-enhanced CT, positron emission tomography-CT (PET-CT), or MRI scans, as well as laboratory examinations (including alpha-fetoprotein (AFP) and alanine aminotransferase (ALT) levels and peripheral blood counts), to monitor adverse events and evaluate compliance. Overall survival (OS) was measured from the start of SRT to death or last follow-up, while progression-free survival (PFS) was from the start of SRT to disease progression or death from any cause. Tumor response was assessed with RECIST v1.1. Local control (LC) was defined as the absence of progression within the irradiated field, encompassing complete response (CR), partial response (PR), or stable disease (SD) of the treated lesions. Conversely, local failure (LF) was defined as progressive disease (PD). Treatment-related toxicities was graded using the Common Terminology Criteria for Adverse Events (CTCAE) v5.0, with 90 days distinguishing acute from chronic toxicity.

### Statistical analysis

Continuous variables were presented as medians and interquartile ranges (IQRs) or ranges, while categorical variables as counts and percentages. OS and PFS were calculated using Kaplan-Meier analysis and compared using the log-rank test. Cox regression identified predictive factors for OS, PFS, and LC, reporting hazard ratios (HR), 95% confidence intervals (CI), and P values. A Forward LR method selected variables for the final multivariate model, retaining those with significance levels below 0.1. Sixteen missing AFP values were imputed using Multiple Imputation by Chained Equations (MICE) with 50 imputations, incorporating key prognostic variables (e.g., tumor size, sites and number of recurrent lesions, and survival status). The nomogram risk model for OS was developed and tested for accuracy using R 4.2.2. Its calibration was visually assessed by comparing actual and predicted survival rates. Internal validation was performed with 1,000 bootstrap resamples. The optimal risk-stratification cut-off value was determined by receiver operating characteristic (ROC) curve analysis and was subsequently used to classify patients into high- and low-risk groups. Kaplan-Meier survival analysis then compared the cumulative survival rates of these groups.

## Results

### Patients and treatment characteristics

A total of 79 patients and 133 treated lesions were included. The median follow-up was 11.4 months (IQR: 6.1-22.8). The median time from transplantation to the first recurrence and subsequent SRT was 6 and 14.4 months, respectively. This interval is due to 31 patients receiving other treatments, like local resection, ablation, or systemic therapy, before SRT. The median OS was 15.1 months (IQR: 7.0-32.9). 46% of patients received concomitant treatment at the time of SRT. Commonly utilized targeted medicines included Sorafenib and Lenvatinib. Chemotherapy regimens contained Irinotecan and Cisplatin in conjunction with Fluorouracil or sequential oral Capecitabine. **Table 1** outlines patient demographics, including 17 patients treated with palliative radiotherapy for symptomatic (predominantly osseous) metastases and 62 receiving curative-intent radiotherapy. For those with multifocal disease receiving radical radiation, non-SRT-targeted lesions were treated with additional local methods like surgical resection or ablation.

**Table 1:** Patient characteristics.

Characteristics	Values
Male, n(%)	74 (94)
Age*	51 (44-55)
KPS*, n(%)	
≥70	74 (93.7)
<70	5 (6.3)
Hepatocirrhosis, n(%)	
HBV	73 (93)
HCV	5 (6)
Schistosome	1 (1)
Pretransplant locoregional therapy, n(%)	
0	26 (33)
1-3	44 (56)
>3	9 (11)
Pretransplant locoregional therapy modality, n	
TACE	38
Surgery	20
Ablation	14
Radiation	2
Transplantation	1
Maximum pretransplant AFP(ng/ml)*	1038 (95.38-3025)
Immediate pretransplant AFP(ng/ml)*	627.5 (14.34-2288)
Posttransplant AFP(ng/ml)*	14.56 (2.97-151.08)
Type of graft, n(%)	
Whole	77 (97)
Right lobe	2 (3)
Time from diagnosis to transplantation(mo)*	3.7 (1.3-11.6)
Time from transplantation to first recurrence(mo)*	6.0 (2.4-12.1)
Time from recurrence to death(mo)*	16 (9.6-29.9)
Time from transplantation to recurrence for SRT(mo)*	11.2 (5.1-19.3)
AFP at recurrence for SRT (ng/ml)*	57.9 (6.1-979.2)
Number of recurrence sites, n(%)	
1-3	63 (80)
>3	16 (20)
Number of recurrent lesions, n(%)	
1-3	18 (23)
4-5	27 (34)
>5	34 (43)
Concomitant systematic therapy, n(%)	36 (46)

IQR, interquartile range; HBV, hepatitis B virus; HCV, hepatitis C virus; AFP, alphafetoprotein.

*median (IQR).

**Figure 1:**
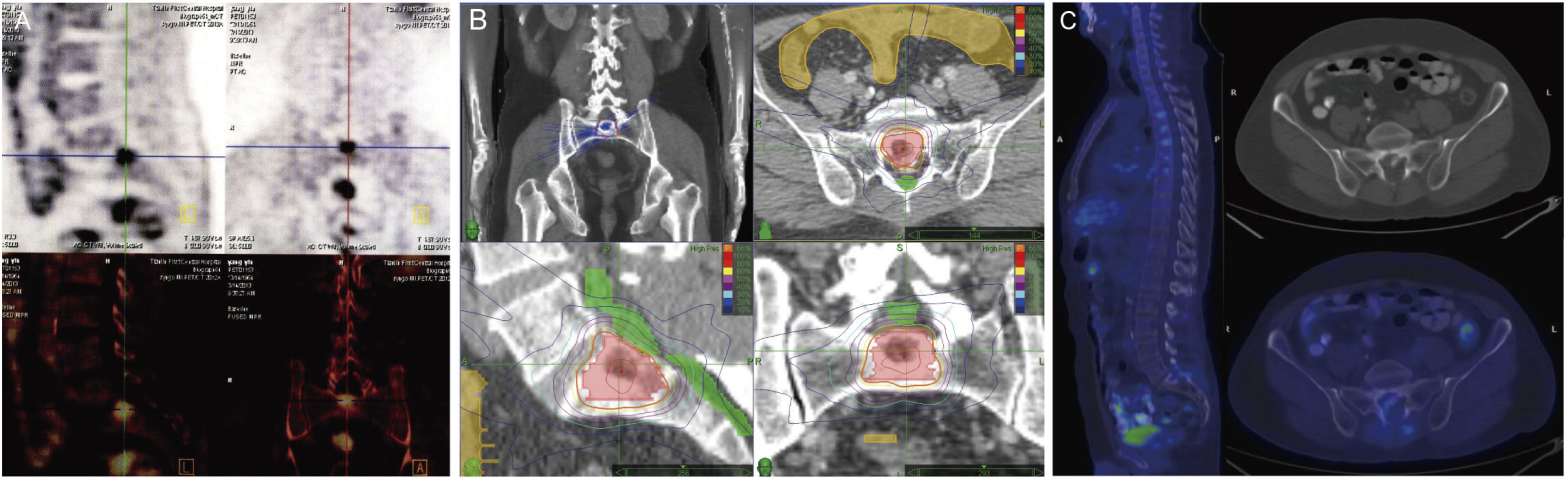
Examples of dose distributions and treatment outcome for one patient. **(A)** PET-CT scan before SRT. **(B)** Treatment plan with 30Gy in three fractions. **(C)** PET-CT scan 18 months after SRT.

Recurrent tumor sites encompassed lungs (35%), the bone (32%), lymph nodes (13%), adrenal glands (11%), grafted livers (6%), the brain (1%) and rare locations such as the spleen, chest wall and diaphragm (2%). The median GTV was 17.3 mL. The largest lesion was a pelvic lymph node with a GTV of 152.3 mL and a prescribed dose of 55Gy/10F. **Table 2** summarizes the anatomical locations, volumetric dimensions, and dose prescription parameters of target lesions. The radiation dose fractionation regimen was established by three board-certified radiation oncologists, with comprehensive consideration of lesion topography, prior radiation, tumor burden, organ-at-risk constraints, and individualized clinical profiles including Karnofsky performance status(KPS). The representative PET-CT scan before (A) and 18 month after (C) SRT, and the corresponding treatment plan (B) for one patient with a sacral vertebral metastasis treated with 30Gy in three fractions (biologically effective dose [BED]_10_ = 60 Gy) are shown in **Figure 1**.

**Table 2:** Characteristics of target lesions.

Site	n(%)	Tumor size(ml)*	Prescribed dose(Gy)*	Number of fractions*	Dose per fraction(Gy)*	BED_10_(Gy)*
Lung	46 (35)	6.5 (0.2-23.7)	45 (15-60)	3 (1-9)	18 (6.67-33)	130.2 (37.5-180)
Bone	43 (32)	22.9 (1.2-118.3)	30 (15-40)	3 (1-5)	10 (7-22)	60 (35.7-89.7)
Lymph node	17 (13)	27.0 (4.2-152.3)	40 (36-55)	5 (3-10)	8 (5.5-13)	79.2 (65.625-102.6)
Adrenal gland	14 (11)	46.9 (14.9-100.1)	40 (35-45)	5 (3-5)	9 (7-15)	85.5 (59.5-112.5)
Liver allograft	8 (6)	11.7 (4.4-57.0)	45 (36-45)	3 (3-5)	13.5 (8-15)	99 (72-112.5)
Brain	2 (1)	20.3 (8.2-32.5)	37.5 (35-40)	5 (5-5)	7.5 (7-8)	65.75 (59.5-72)
Other (Spleen/Chest wall/Diaphragm)	3 (2)	74.0 (50.5-131.3)	40 (40-55)	5 (3-9)	8 (6.1-15)	88.61 (72-112.5)

*median (range).

### LC and predictors for LF

**Figure 2:**
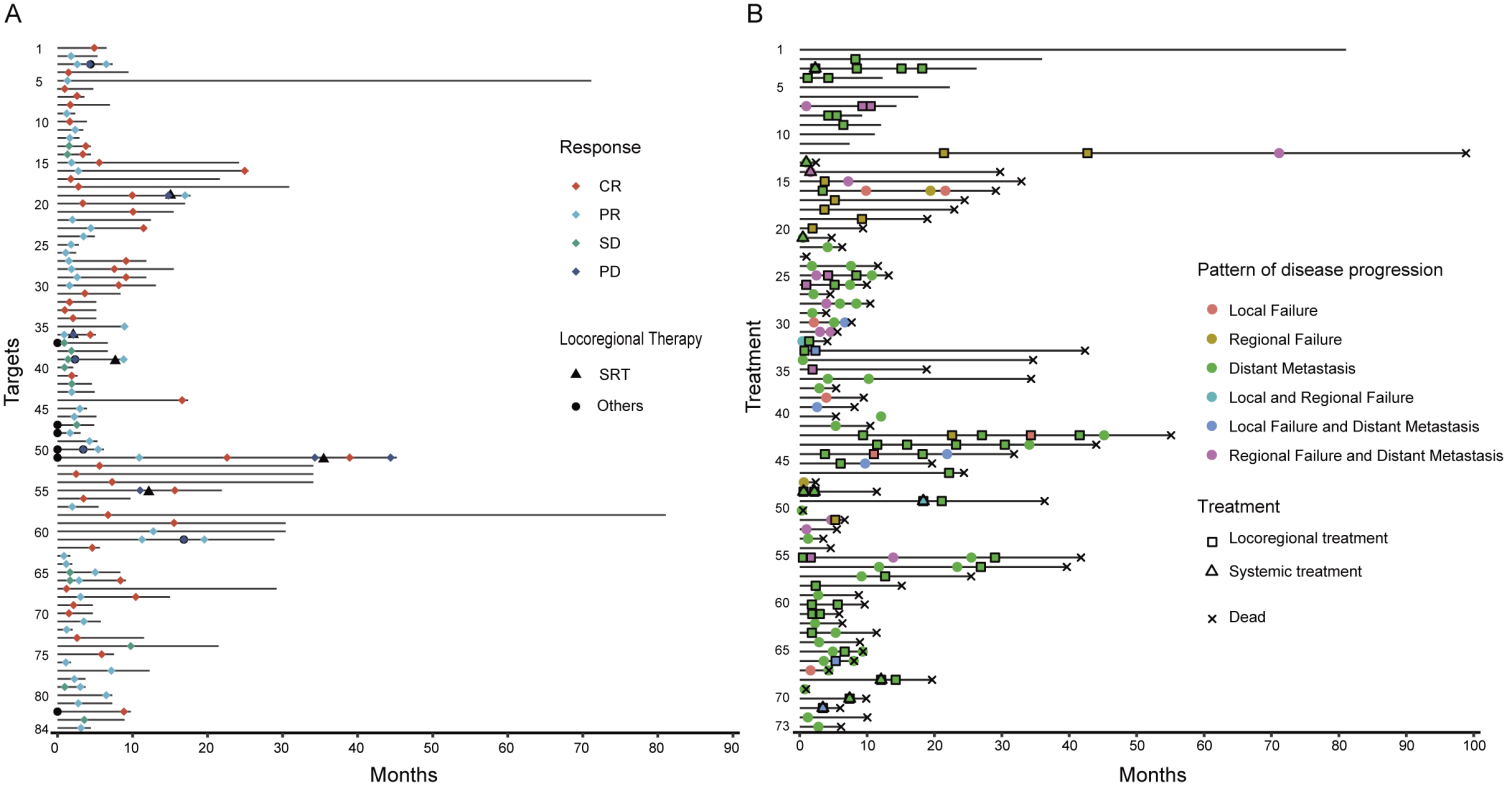
Swimming plots of local response by lesion **(A)** and disease progression by treatment **(B)**.

**Figure 3:**
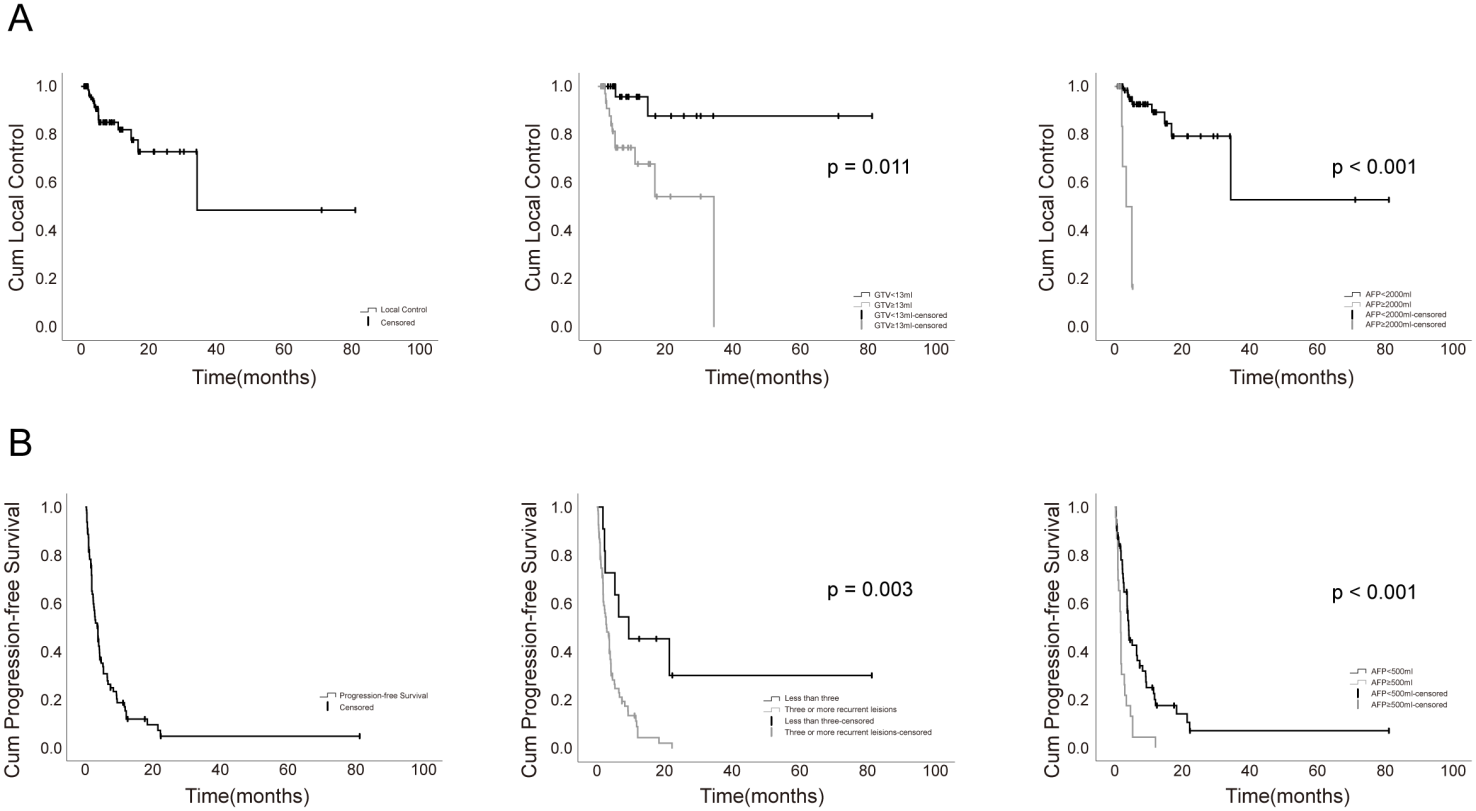
Actuarial local control and progression-free survival (PFS) of patients. **(A)** Overall local control And local control depending on Gross target volume (GTV), pre-treatment AFP. **(B)** Overall PFS and PFS depending on pre-treatment AFP, the number of recurrent lesions.

The overall local control rate (LCR) was 90.5%, with specific recurrences showing LCRs of 92.8% for adrenal gland, 91.3% for lung, 90.7% for bone, 88.2% for lymph nodes, and 87.5% for liver. CR was achieved in 47.6% of target lesions, while PR, SD, and PD rates were 34.5%, 8.3%, and 9.5%, respectively. Median times to PR, SD, and CR were 3.8, 5.5, and 9.7 months, with baseline GTVs of 6.6 mL for CR, 20.3 mL for PR, and 31.5 mL for SD. **Figure 2A** displays local responses of 84 imaging-mensurable lesions. Multivariate Cox analysis identified per-lesion GTV ≥ 13 mL (HR = 4.564, 95% CI: 0.914-22.800, *p* = 0.064) and pre-treatment AFP ≥ 2,000 ng/mL (HR = 11.717, 95% CI: 2.939-46.716, *p* < 0.001) as predictors of LF (**Figure 3A**). Failed locoregional therapies before SRT were notably poor in univariate analysis (HR = 5.907, 95% CI: 1.725-20.227). All 17 patients with severe symptoms from intracranial and bone metastases experienced symptom relief after SRT.

### PFS and predictors of PFS

In 79 patients, the first progression after SRT was mainly due to distant metastases (54%), with a median progression time of 3.7 months. PFS data for 73 patients is shown in **Figure 2B**. Cox regression analysis identified that having three or more recurrent lesions (HR = 3.078, 95% CI: 1.364-6.949, *p* = 0.007) and pre-treatment AFP levels of 500 ng/mL or higher (HR = 2.532, 95% CI: 1.494-4.290, *p* = 0.001) independently predicted disease progression (**Figure 3B**). SRT targeting the initial recurrence site was associated with improved PFS (HR = 0.900, 95% CI: 0.819-0.989), whereas total GTV≥40 mL correlated with inferior PFS (HR = 1.891, 95% CI: 1.150-3.108) in univariate analysis (**Table 3**).

**Table 3:** Univariate and multivariate predictors of PFS.

Variable	Univariate			Multivariate		
	HR	95% CI	*P*	HR	95% CI	*P*
Age (per SD)	1.058	0.841-1.332	0.631			
KPS						
<70	ref	–	–			
≥70	0.424	0.166-1.081	0.072			
Number of recurrence sites	1.160	0.943-1.427	0.159			
Site of recurrent lesion						
Liver allograft	ref	–	–			–
Lung	1.040	0.952-1.137	0.382			
Bone	1.013	0.937-1.095	0.751			
Lymph Node	0.984	0.904-1.072	0.714			
Adrenal Gland	1.052	0.931-1.189	0.416			
Brain	1.055	0.898-1.239	0.513			
Number of recurrent lesions						
<3	ref	–	–	ref	–	–
≥3	3.392	1.507-7.634	0.003	3.078	11.364-6.949	0.007
Oligo-recurrence/progression at SRT	0.524	0.238-1.156	0.110			
Previous Systemic Therapy						
No previous therapy	ref	–	–			
Chemotherapy, cycle	1.097	1.005-1.197	0.038			
Targeted therapy, mo	0.993	0.972-1.015	0.550			
Concurrent Systemic Therapy	1.055	0.961-1.160	0.262			
Target lesion as first recurrence	0.900	0.819-0.989	0.029			
AFP at recurrence for SRT						
<500 ng/ml	ref	–	–	ref	–	–
≥500 ng/ml	2.776	1.637-4.708	<0.001	2.532	1.494-4.290	0.001
Total GTV						
<40 ml	ref	–	–			
≥40 ml	1.891	1.150-3.108	0.012			

### OS and predictors of mortality by NOMOGRAM

**Figure 4:**
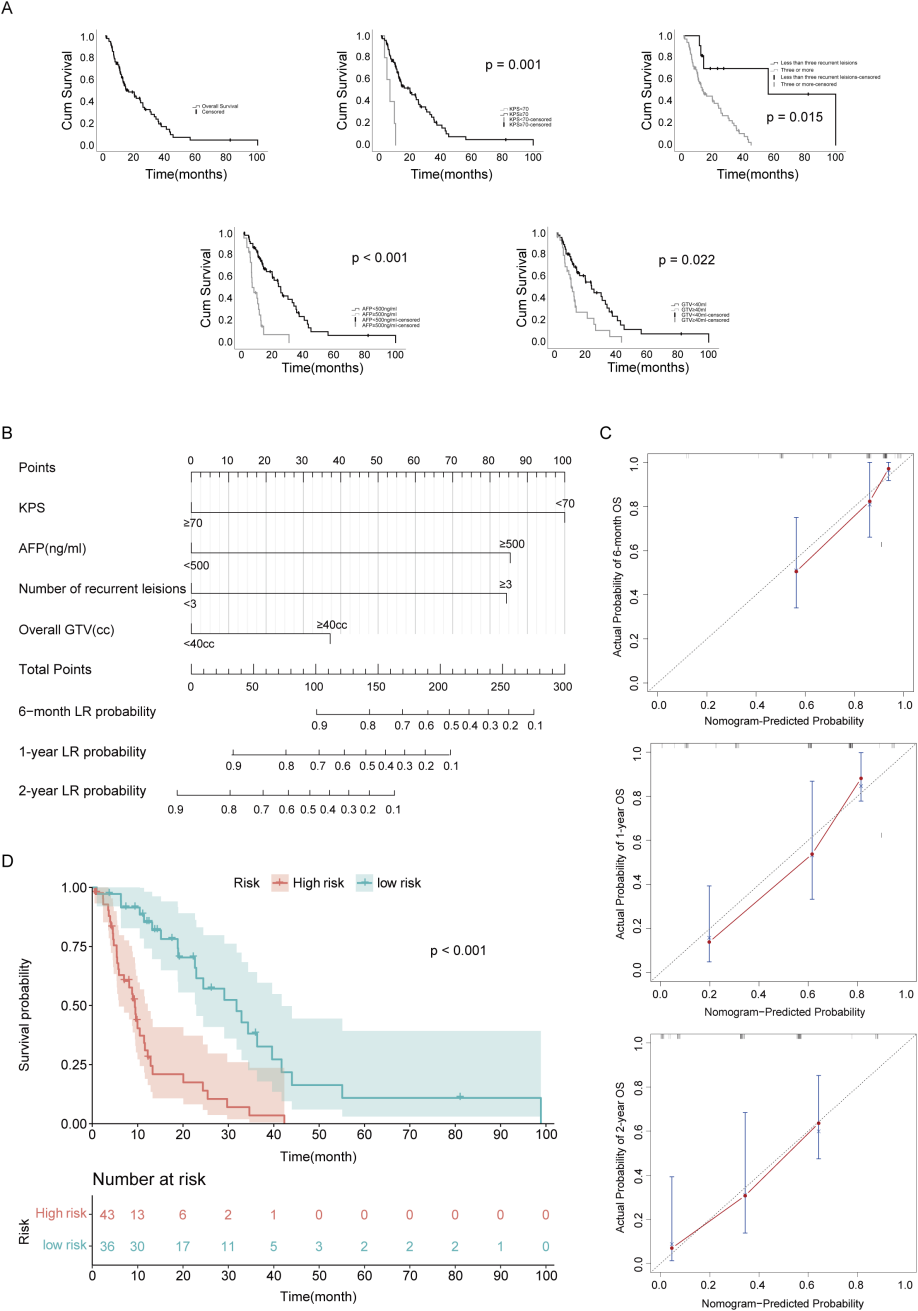
Actuarial overall survival (OS) of patients and a novel nomogram to predict OS. **(A)** OS in general and OS depending on KPS, the number of recurrent lesions, pre-treatment AFP and overall GTV. **(B)** The nomogram provides a visual point system based on the combination of four characteristics to estimate the survival of 6-month,1-year and 2-year. **(C)** Internal calibration curves of 6-month,1-year and 2-year OS for HCC patients received SRT after recurrence. Dotted line, the ideal reference line where predicted probabilities would match the observed survival rates; red dots, calculated by bootstrapping (resample: 1000), represent the performance of the nomogram. **(D)** OS curves of patients with different risks stratified by the nomogram. Cum, cumulative.

The median OS was 15.1 months (IQR 7.0-32.9), with 6-month, 1-year, and 2-year OS rates of 78.9%, 57.1%, and 38.9%, respectively. Multivariate Cox regression analysis identified four independent prognostic factors for OS: KPS ≥70 (HR = 0.171, 95% CI: 0.062-0.470, *p* = 0.001), presence of ≥3 recurrent lesions (HR = 4.465; 95% CI: 1.330-14.989, *p* = 0.015), pretreatment AFP ≥500 ng/mL (HR = 4.520, 95% CI: 2.410-8.477, *p* < 0.001), and total GTV ≥40 mL (HR = 1.927; 95% CI: 1.100-3.378, *p* = 0.022) (**Table 4**; **Figure 4A**).

**Table 4:** Univariate and multivariate predictors of OS.

Variable	Univariate			Multivariate		
	HR	95% CI	*P*	HR	95% CI	*P*
Age (per SD)	1.041	0.821-1.320	0.740			
KPS						
<70	ref	–	–	ref	–	–
≥70	0.176	0.065-0.476	0.001	0.171	0.062-0.470	0.001
Number of recurrence sites	1.226	0.990-1.518	0.062			
Site of recurrent lesion						
Liver allograft	ref	–	–			
Lung	1.000	0.972-1.029	0.999			
Bone	1.009	0.983-1.035	0.519			
Lymph Node	1.869	1.006-3.278	0.029			
Adrenal Gland	1.012	0.979-1.047	0.484			
Brain	5.152	1.900-13/971	0.001			
Number of recurrent lesions						
<3	ref	–	–	ref	–	–
≥3	5.854	1.779-19.263	0.004	4.465	1.330-14.989	0.015
Oligo-recurrence/progression at SRT	0.522	0.234-1.165	0.112			
Previous Systemic Therapy						
No previous therapy	ref	–	–			
Chemotherapy, cycle	1.123	1.017-1.239	0.021			
Targeted therapy, mo	0.992	0.967-1.019	0.567			
Concurrent Systemic Therapy	1.009	0.983-1.035	0.498			
Target lesion as first recurrence	0.613	0.358-1.048	0.073			
AFP at recurrence for SRT						
<500 ng/ml	ref	–	–	ref	–	–
≥500 ng/ml	4.673	2.508-8.706	<0.001	4.520	2.410-8.477	<0.001
Total GTV						
<40 ml	ref	–	–	ref	–	–
≥40 ml	2.254	1.300-3.910	0.004	1.927	1.100-3.378	0.022

Based on these predictors, we developed a prognostic nomogram (**Figure 4B**) that calculates 6-month, 1-year, and 2-year survival probabilities through summation of assigned scores. The model demonstrated good discrimination with a bootstrap-validated concordance index (C-index) of 0.760 (95% CI, 0.696-0.824). Calibration was assessed by plotting predicted versus observed survival probabilities, which showed excellent agreement across all time points, with calibration curves closely aligning with the 45-degree reference line and no significant deviations observed (**Figure 4C**). ROC analysis identified an optimal prognostic cutoff score of 84 points. At this threshold, the sensitivity was 63.2% (95% CI, 56.0%–74.0%) and specificity was 63.6% (95% CI, 56.5%–73.5%), indicating moderate discriminative ability. Stratifying patients by this threshold revealed significantly divergent survival curves (*p* < 0.001), with the high-risk group (total score >84) exhibiting inferior survival compared to the low-risk group (≤84 points) (**Figure 4D**).

### Treatment toxicity

The treatment demonstrated favorable tolerability across all patients. Acute toxicities comprised 13 grade 1 events (fatigue [n=7], nausea [n=3], diarrhea [n=3], fever [n=1]) and 5 grade 2 events (vomiting [n=2], leukopenia [n=2], esophagitis [n=1]). Notably, no grade ≥3 toxicities were observed throughout the treatment course.

## Discussion

Expanding liver transplantation criteria has increased post-transplant HCC recurrence. Current management is debated, relying on expert opinion and non-randomized studies (13, 14), complicated by recipients’ immunocompromised status and infection risk (14, 15). Safer, effective treatments are needed. While SRT has demonstrated 90-95% 2-year local control rates in primary HCC (16, 17) and comparable efficacy to RFA without vascular proximity or tumor size limitations (14, 18, 19), its application in post-transplant recurrence remains under-explored. This study assesses SRT’s effectiveness in this context and aims to develop evidence-based selection criteria for high-risk patients.

Our analysis demonstrates that SRT provides robust local control for post-transplant recurrences, with a median OS of 15.1 months that compares favorably to historical benchmarks of 10–13 months (6, 20, 21). This improvement is likely due to better baseline performance and aggressive local treatments for recurrences. Fewer than three recurrent lesions and pretreatment AFP levels below 500 ng/ml were identified as independent predictors of longer OS and PFS. Additional favorable factors included a KPS of 70 or higher and total GTV under 40 ml, indicating limited tumor burden and less aggressive behavior. The significantly superior outcomes observed in low-risk patients—defined by the presence of ≤1 risk factor (≥3 lesions or total GTV ≥40 mL)—strongly validate the therapeutic value of local ablative approaches like SRT in this subset.

The lower CR rate (47.6%) compared to the higher PR (34.5%) likely results from the short 6.6-month median follow-up, as CR takes longer to achieve (median 9.7 months) than PR (3.8 months). Of five secondary recurrence cases treated with SRT, four showed an objective response, while one had disease progression. This may be due to dosimetric limitations in re-irradiating areas and tumor radioresistance. Multivariate analysis confirmed known predictors of LF, such as high AFP levels and larger tumor volumes, aligning with previous findings (22, 23). The lack of a clear link between BED_10_ (≥60Gy) and local control is likely due to various factors, such as anatomical differences, a wide range of tumor volumes (0.2-131.3 mL) affecting radiobiology, mixed treatment intents, and post-transplant immunosuppression altering tissue responses, all of which overshadow theoretical dose benefits.

Although local therapy generally confers limited survival benefit in high-risk patients, our subsidiary analysis of 17 cases with bulky symptomatic disease demonstrated that SRT can effectively achieve meaningful palliation by reducing tumor burden and alleviating debilitating symptoms, thereby improving quality of life. Interpretation of our survival outcomes, particularly OS, must account for two key limitations. The cohort included patients treated with both curative and palliative intent, introducing potential heterogeneity. Furthermore, patients often received other local and systemic therapies alongside SRT, which could not be fully adjusted for in this retrospective analysis. Consequently, the OS results reflect the net effect of a multimodal treatment strategy rather than the efficacy of SRT in isolation.

The safety profile in our cohort supports the use of SRT in this vulnerable population. Most toxicities were mild and temporary, with two cases of grade 2 leukopenia due to targeted irradiation of large bone metastases (2/43, 4.7%). SRT-induced esophagitis and diarrhea were manageable, confirming that with adapted dosing and PTV margins, SRT remains a safe and viable option even in the palliative setting.

Unlike previous studies that associated intrahepatic recurrence with poor prognosis (20, 24)—often due to limited treatment options after recurrence—our study found no significant association between intrahepatic lesions at the time of SRT and worse survival. This discrepancy may be explained by the efficacy of SRT in controlling localized liver disease or the limited sample size of patients with intrahepatic involvement in our cohort. Further larger prospective studies are needed to clarify the influence of recurrence patterns on prognosis in patients undergoing SRT.

A key contribution of this study is the development of a predictive nomogram for survival risk stratification in patients with post-transplant HCC recurrence. In addition to its statistical performance, the nomogram provides clinical guidance for personalized monitoring and treatment—such as closer surveillance during the high-risk first year, monitoring AFP trends, paying attention to lung and bone metastases, and enabling timely SRT intervention. Framing the model as a predictive algorithm reflects the move toward data-driven, individualized oncology.

AFP plays a central role in this approach. As a significant prognostic factor in our study, it also serves as an early marker of recurrence (25, 26), often rising before imaging changes. Its dual function supports its inclusion in the nomogram and highlights its value from surveillance to treatment planning. Although Des-γ-carboxy-prothrombin (DCP) and other key liver function parameters were not included in our cohort, they may offer added value in future models as supplements among AFP non-secretors (25, 27).

SRT should be viewed as a downstream part of surveillance-based care. Since recurrence often occurs within a narrow time window, early detection using biomarkers and imaging can guide timely treatment. Incorporating SRT into this context supports its role in personalized post-transplant management.

Emerging evidence suggests that SRT can potentiate anti-tumor immunity and synergize with immunotherapy (28, 29). Given the established efficacy of immune checkpoint inhibitors in controlling post-transplant recurrence (30), combining SRT with immunotherapy may represents a highly promising avenue for future study. Although not explored in the present cohort, our safety and efficacy data provide a foundational rationale for subsequent trials investigating such combinatorial approaches.

This study has several limitations. Its retrospective design and relatively small sample size limit the statistical power for reliable subgroup analyses and may introduce selection bias. Consequently, the constructed nomogram currently demonstrates suboptimal sensitivity and specificity, its performance requires further improvement and validation. The extended enrollment period inevitably led to inconsistencies in clinical management and data collection. Additionally, as a single-center study, the nomogram lacks external validation, which may affect its generalizability. Finally, a longer follow-up duration is required to fully assess the long-term efficacy and late toxicity profiles of SRT in this population.

## Conclusion

SRT provides effective control of post-transplant HCC recurrence for both hepatic and extrahepatic lesions. A novel four-factor nomogram enables individualized survival prediction and supports optimal patient selection, reinforcing the role of SRT within a personalized, biomarker-informed treatment strategy.

## Data Availability

The original contributions presented in the study are included in the article/supplementary material. Further inquiries can be directed to the corresponding authors.
